# Sleep problems among adolescents within child and adolescent mental health services. An epidemiological study with registry linkage

**DOI:** 10.1007/s00787-020-01676-4

**Published:** 2020-11-07

**Authors:** Mari Hysing, Ove Heradstveit, Allison G. Harvey, Sondre Aasen Nilsen, Tormod Bøe, Børge Sivertsen

**Affiliations:** 1 Department of Psychosocial Science, Faculty of Psychology, University of Bergen, Bergen, Norway; 2 Regional Centre for Child and Youth Mental Health and Child Welfare, NORCE Norwegian Research Centre, Bergen, Norway; 3 Center for Alcohol & Drug Research, Stavanger University Hospital, Stavanger, Norway; 4 Department of Health Promotion, Norwegian Institute of Public Health, Bergen, Norway; 5 Department of Research & Innovation, Helse-Fonna HF, Haugesund, Norway; 6 Department of Mental Health, Norwegian University of Science and Technology, Trondheim, Norway; 7 Department of Psychology, University of California, Berkeley, USA

**Keywords:** Insomnia, Short sleep duration, DSWPD, Psychiatric disorders, Adolescence

## Abstract

Sleep problems are prevalent among adolescents, especially among those diagnosed with mental health disorders. There is insufficient knowledge about sleep among adolescents within child and adolescent mental health services (CAMHS) in comparison to the general population. The data are drawn from the youth@hordaland study, a large population-based study conducted in 2012, linked to the Norwegian Patient Registry (NPR) (*n* = 9077). Psychiatric disorders were based on clinical diagnoses from the NPR, while insomnia, delayed sleep–wake-phase disorder (DSWPD), and other sleep problems/patterns were assessed by self-report questionnaires from youth@hordaland. The prevalence of diagnosed sleep disorders among adolescents seeking mental health services was 0.6%, yielding an estimated prevalence of 0.07% of the population. However, questionnaire-based measurement of insomnia from the youth@hordaland study indicated that insomnia was highly prevalent across disorders in comparison to a reference group of adolescents who were not within mental health care. Insomnia ranged from 29% among adolescents diagnosed with ADHD (PR = 1.79; 95% CI 1.41–2.29) to 48% among adolescents diagnosed with depression (PR = 2.53, 95% CI 2.19–2.92). All diagnostic groups had a mean sleep efficiency below (85%), indicating poor sleep quality. Insomnia, delayed sleep-phase wake disorder, and poor sleep efficiency were confirmed as transdiagnostic sleep problems across psychiatric disorders. In addition, some disorder-specific patterns emerged, such as a higher prevalence of insomnia among adolescents with depression, and DSWPS among adolescents with conduct disorder. This underscores the need for treating sleep problems in CAMHS, and transdiagnostic treatment approaches are warranted.

## Introduction

Sleep problems are prevalent among adolescents after puberty [1]. Sleep during this developmental stage is characterized by short sleep duration on weekdays, a delayed sleep phase, and a high prevalence of insomnia [2, 3]. Adolescents with mental health problems have an even higher prevalence of sleep problems across diagnosed disorders [4].

Sleep problems are commonly listed as core symptoms of many psychiatric disorders, including major depressive disorder (MDD) and generalized anxiety disorder (GAD) [5]. Sleep problems may be a causal factor in the development of such psychiatric disorders [6], but also a consequence of these [7]. This bidirectional perspective is in sharp contrast to the previously often held belief that sleep problems were secondary to a psychiatric disorder [8], and thus rarely diagnosed or targeted in mental health care interventions. This change in perspective is also manifested in the transition from DSM-IV to DSM-5, where the previous distinction between insomnia *with* and *without* a co-existing mental disorder is no longer included in the latest revision [5, 9, 10]. This shift has also led to a greater emphasis on diagnosing and treating sleep problems when these co-occur alongside a mental health disorder.

Still, we know little about the prevalence of sleep problems among adolescents seeking help from child and adolescent mental health services (CAMHS). One study that has shed light on sleep patterns among adolescents with psychiatric disorders, is the National Comorbidity Study from 2000 to 2004 that is based on a nationally representative sample recruited from the USA. This study assessed sleep patterns, not sleep disorders, across several psychiatric diagnoses among over 10,000 adolescents aged 13 to 18 years. The results indicated later weeknight bedtime, shorter weeknight sleep duration, greater weekend bedtime delay, and both short and long periods of weekend oversleep were associated with increased odds of mood, anxiety, substance use, and behavioral disorders [4]. A Norwegian study found a higher level of sleep problems assessed by five items from the Youth Self Report Questionnaire in the clinical sample of adolescents attending specialty health care clinic (31%), relative to a community sample (5%) [11]. The importance of increasing our attention to sleep problems in mental health care services in general was also demonstrated in a study of adults receiving treatment in public mental health service clinics, where sleep problems were an independent predictor of impaired functioning and fewer benefits from treatment [12].

Sleep problems co-occurring with psychiatric disorders may take many forms including insomnia [7, 13, 14], circadian rhythm disorders, and a short sleep duration [15]. Although sleep problems can be considered a transdiagnostic phenomenon [16], the prevalence and patterns of sleep problems may vary across different psychiatric disorders. Employing a unique linkage approach whereby official data from registries that record mental illness based on clinician diagnoses, provides opportunity to compare adolescents connected to mental health care versus adolescents not connected to care. The linkage also allows for similar detailed sleep assessment for both groups. Sleep patterns and sleep problems differ by gender [1, 3], and similarly there are gender differences in the prevalence of psychiatric disorders [17, 18], and thus gender needs to be taken into account when investigating the association.

The overall aim of the current study was to estimate the prevalence of key sleep and circadian disorders (insomnia, DSWPD) and key sleep parameters (bed time, rise time, sleep-onset latency, total sleep time, time in bed and sleep efficiency) in adolescents within mental health care (clinical group) in comparison to a reference group from the general population adjusting for gender.

## Methods

### Procedure

This population-based study used data from the youth@hordaland-survey of adolescents in Hordaland County in Western Norway. All adolescents born between 1993 and 1995 were invited in 2012 with an aim to reach all adolescents in late adolescence and high school age. The main aim of the survey was to assess the prevalence of mental health problems and service use in adolescents. Adolescents in upper secondary education received study information via e-mail, and one classroom school hour was allocated for completing the questionnaire. Those not in school received information by postal mail to their home address. The questionnaire was web-based. It covered a broad range of mental health issues, daily life functioning, use of health care and social services, and demographic characteristics, as well as a request for permission to obtain school data and link the information with national health registries. For those who consented to registry linkage, the data from the youth@hordaland study were linked to the National Patient Registry (NPR), the official registry for specialist health care in Norway and provide data from specialized child and adolescent mental health care services. The linkage was performed by the registry owner at the Norwegian Directorate of Health and a deidentified dataset consisting of data from the NPR and the youth@hordaland was used in the present study.

### Sample

All adolescents born between 1993 and 1995 were invited (*N* = 19,430) to participate in the epidemiological study during the first months of 2012, of which 10,257 agreed, yielding a participation rate of 53%. The validity of sleep data was assessed manually, with participants providing obvious invalid responses being omitted from further analyses. Invalid responses included 1) sleep-onset latency (SOL) or wake-after-sleep onset (WASO) more than 12 h, 2) SOL + WASO longer than time in bed (TIB), and 3) negative values of sleep duration and sleep efficiency. The adolescents that consented to linkage to registries (*n* = 9411) and had valid sleep data are included in the present study (*n* = 9007).

#### Representativeness

In a study on an earlier linkage between NPR and youth@hordaland study, those who did not consent to the linkage had more self-reported conduct problems, were somewhat older, and had a slightly increased rate of high-level alcohol consumption. However, the differences were all small in magnitude, with Cohen’s *d* effect sizes ranging from 0.09 to 0.26 [19].

### Instruments

#### Socio-demographics

Gender and date of birth were identified through personal identity number in the Norwegian National Population Register. Exact age was estimated by calculating the interval of time between the date of birth and date of participation. Socioeconomic status (SES) was assessed both by perceived economic well-being and parental education. Perceived economic well-being was reported with three response options: “poorer than others”, “equal to others”, and “better than others.” Maternal and paternal education were reported separately with three response options: “primary school”, “secondary school”, and “college or university”.

#### Sleep measures

Insomnia was operationalized in alignment to the DSM-5 criteria [5]. Difficulties initiating and maintaining sleep (DIMS) were rated on a three-point Likert-scale with response options “not true”, “somewhat true” and “certainly true”. If confirmed (i.e., “somewhat true” or “certainly true”), adolescents were then asked how many days per week they experienced problems either initiating or maintaining sleep. Adolescents also provided information on the duration of DIMS. A joint question on tiredness/sleepiness was rated on a three-point Likert-scale with response options “not true”, “somewhat true” and “certainly true”. If confirmed, adolescents then reported the number of days per week they experienced sleepiness and tiredness, respectively. To meet the DSM-5 criteria for insomnia, adolescents had to report DIMS at least three times a week, with a duration of three months or more, as well as tiredness or sleepiness at least three days per week.

Delayed sleep–wake-phase disorder (DSWPD) was assessed by the following questions: “At what time do you usually go to bed?”, “How much time does it take before you fall asleep (hours and minutes)?”, “When do you usually get out of bed in the morning?”, “How many nights per week do you have difficulties falling asleep (0–7)?”, “How many nights per week do you have problems with nightly awakenings (0–7)?”, “How often do you oversleep (“never”, “seldom”, “sometimes”, “mostly”, “always”)?”. The participants provided sleep data separately for weekdays and weekends. No information regarding the time frame of these symptoms was available. To establish a proxy for assessing DSWPD (as close as possible given the available sleep items) in line with the International Classification of Sleep Disorders-2 (American Academy of Sleep Medicine 2005), we employed the following criteria (as specified in Johnson et al. published in Pediatrics): (1) minimum 1-h shift in sleep-onset AND wake times from the weekdays to the weekend, (2) complaint of frequent (≥ 3 days per week) difficulty falling asleep, (3) report of little or no (≤ 1 day per week) difficulty maintaining sleep, and (4) frequent difficulty awakening (oversleep “sometimes” or more often).

Self-reported usual bedtime and rise time were indicated in hours and minutes using a scroll down menu; data were reported separately for weekdays and weekends. Time in bed (TIB) was calculated as the difference between bedtime and rise time. Sleep-onset latency (SOL) and wake-after-sleep onset (WASO) were indicated in hours and minutes, and sleep duration was defined as TIB minus SOL and WASO. Sleep efficiency was calculated as sleep duration divided by TIB multiplied by 100 (reported as a percentage; higher scores reflecting greater sleep efficiency). Sleep efficiency was analyzed as a continuous variable, with less than 85% as the cut-off for indications of poor sleep quality in accordance with Lacks and Morin [20]

#### Psychiatric disorders

Psychiatric disorders are based on Axis 1 diagnoses from the Norwegian Patient Registry (NPR). The psychiatric disorders were coded in accordance with Axis 1 in the ICD-10 diagnostic manual by clinicians in the CAMHS, and we assigned all the diagnoses into broader diagnostic categories included in the present study: depression, anxiety, attention-deficit/hyperactivity disorder (ADHD), conduct disorder, trauma-related disorder, autism, eating disorder, psychotic disorders. Sleep diagnoses (F510 Non-organic insomnia and non- organic disorder of the sleep–wake cycle) were also included. A range of less common psychiatric diagnoses were categorized as ‘other psychiatric diagnoses’ but was not used in these analyses due to a large conceptual heterogeneity [19].

The timing of the contact with CAMHS was categorized into contact initiated before, at the same time and after the youth@hordaland.

### Ethics

The study was approved by the Regional Committee for Medical and Health Research Ethics (REC) in Western Norway (2011/811/REK Vest) and NSD (371,974 and 259,631).

Following the regulations from the REC and Norwegian health authorities, adolescents aged 16 years and older can make decisions regarding their health (including participation in health studies), and thus gave consent themselves to participate in the current study and for the linkage to registries. Parents/guardians have the right to be informed, and in the current study, and all parents/guardians received information about the study in advance.

### Statistical analyses

Independent samples *t*-tests and chi-squared tests were used to examine differences in demographic variables for the adolescents in the clinical sample, i.e. those within the CAMHS (i.e. the clinical group) and in the reference group (i.e. those not in contact with CAMHS). The various psychiatric diagnosis in the clinical group was all compared to the reference group on insomnia and DSWPD using log-link binomial regression analysis. Being similar to how risk ratios (RR) are calculated, the estimates are presented as prevalence ratios (PR) given the cross-sectional nature of this study. Similarly, the sleep parameter in adolescents across diagnosis in the clinical group was compared to adolescents in the reference group by computing estimated marginal means (EMM), adjusting for gender.

## Results

### Characteristics of the sample

The total sample (*n* = 9077) consisted of 53.4% girls, and the mean age was 17.4 years (SD 0.83), range 16–19 years. There were significantly more girls than boys in the clinical group compared to the reference group, while no significant age difference was observed (see Table 1 for details).

**Table 1:** Demographic characteristic of the linked youth@hordaland and the Norwegian Patient Registry (NPR)-dataset and separately for those that have and have not been in contact with CAMHS

	Total (9077)	CAMHS contact (911)	Non-contact (8166)
Girls % (*n*)	53.4%	60.3% (549)	52.7% (4301)
Age in years, mean (SD)	17.4 (.83)	17.3 (.83)	17.4 (.83)
Highest education in the family			
Primary	4.1% (364)	6.1% (55)	3.8% (309)
Secondary	31% (268)	29.8% (268)	31,1% (2510)
College/university	46.0% (4113)	37.8% (339)	46.9% (3774)
Don’t know	18.9% (1695)	26.4% (237)	18.1% (1458)
Insomnia	19.2% (1980)	34.4% (329)	17.7% (1651)
Delayed sleep phase	1.9% (175)	4.0% (37)	2.1% (212)

In all, 911 participants (10%) in youth@hordaland had been in contact with CAMHS and comprised the clinical group. Among these, two-thirds (66.4%) had been in contact before the onset of this epidemiological health survey, 24.8% were in contact during the data collection period, while 8.8% initiated the contact after the health survey. Among the participants in the clinical group, only six adolescents were registered as having a sleep disorder diagnosis (0.6% of people connected with services).

### Insomnia and delayed sleep in adolescents with psychiatric disorders compared to a reference group

Prevalence and PR for insomnia and DSWPD for adolescents with a psychiatric diagnosis in the clinical sample, in comparison to the reference group, are presented in Table 2. In the reference group, 16.7% presented with insomnia, while in the clinical group, the prevalence ranged from 28.7% for ADHD (PR: 1.79, CI 95% [1.41–2.29]) to 48% for depression (PR: 2.53, CI 95% [2.19–2.92]). DSPS was present for 2.9% of the reference group, while the prevalence among those with psychiatric disorders ranged from 0% for eating disorders to 20% for the conduct disorder group (PR: 7.28, CI 95% [3.52–15.05]).

**Table 2:** Prevalence of insomnia and delayed sleep phase and the prevalence ratios (PR) according to psychiatric diagnosis

Diagnosis from the NPR	Insomnia DSM-5 from the youth@hordaland			Delayed sleep–wake-phase syndrome (DSWPD)from the Youth@hordaland		
	%	PR*	95% CI	%	PR	95% CI
Not in contact with CAMHS (ref) (*n* = 8166)s	16.7%	–	–	2.9%	–	–
Depression (*n* = 221)	48.0%	2.53	2.19–2.92	7.4%	2.38	1.46–3.89
Anxiety (*n* = 167)	31.7%	1.73	1.39–2.17	5.6%	1.82	0.95–3.49
ADHD (*n* = 160)	28.7%	1.79	1.41–2.29	9.0%	3.16	1.89–5.29
Conduct (*n* = 30)	40.0%	2.64	1.74–3.99	20.0%	7.28	3.52–15.05
Trauma (*n* = 77)	40.3%	2.14	1.64–2.80	9.2%	2.94	1.44–6.01
Psychosis (*n* = 22)	45.5%	2.33	1.49–3.67	9.1%	**	**
Autism (*n* = 51)	29.4%	2.07	1.37–3.14	6.0%	**	**
Eating disorder (*n* = 56)	42.9%	2.05	1.51–2.79	0%	**	**

*Adjusted for gender

**Analyses were not performed when cells had fewer than five cases

### Sleep parameters among adolescents with psychiatric disorders compared to a reference group

**Fig. 1:**
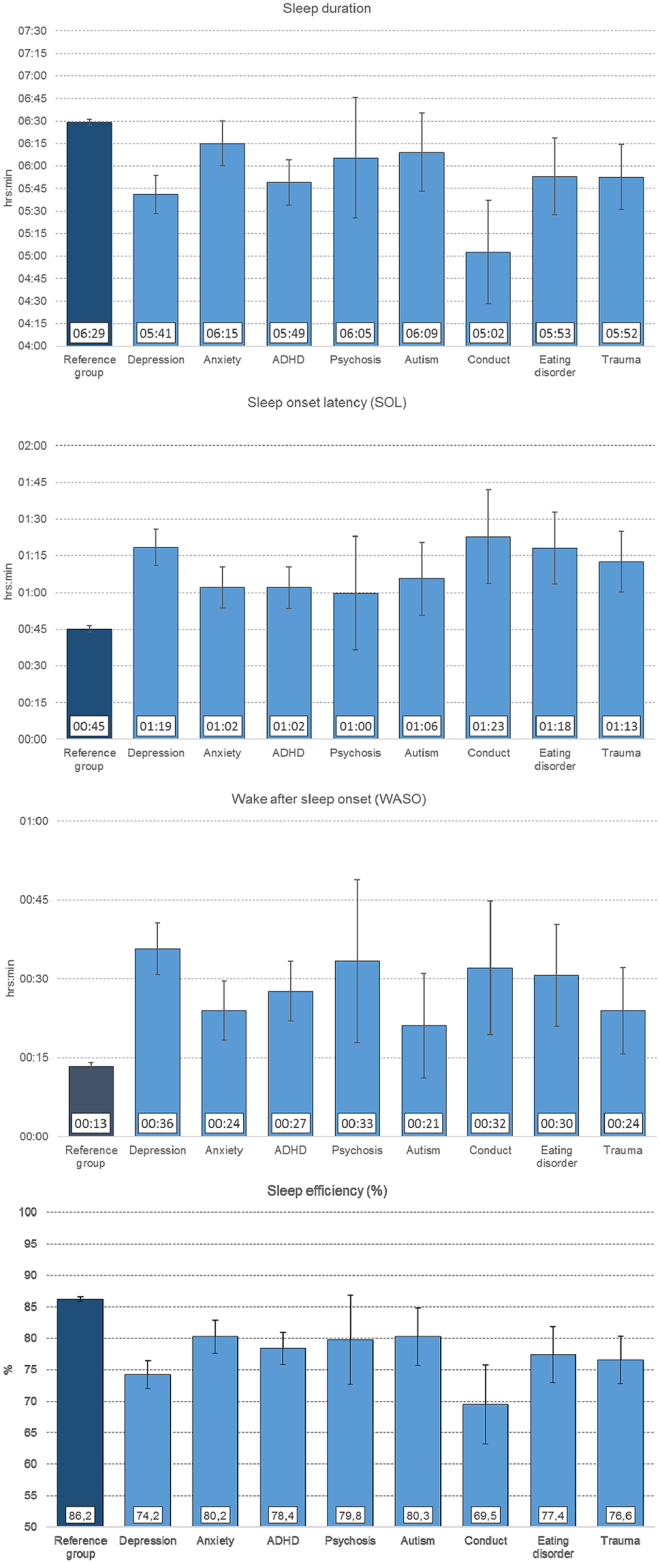
Sleep duration, sleep-onset latency, wake-after-sleep onset and sleep efficiency for adolescents with a diagnosed disorder of depression, anxiety, ADHD, psychotic disorders, autism, conduct, eating disorder and trauma in comparison to a reference group

Estimates of sleep duration, SOL, WASO, and sleep efficiency (adjusting for gender) are displayed in Fig. 1. Compared to the reference group, adolescents diagnosed with depression, ADHD, conduct disorder (ps < 0.001), trauma and eating disorder (*p* < 0.05) had significantly shorter sleep duration. SOL was significantly longer among adolescents with depression, anxiety, ADHD, conduct disorder, trauma, eating disorder, as well as autism (*p* < 0.001) compared to the reference group. With regard to WASO, adolescents with depression, anxiety, ADHD, eating disorder, conduct disorder (ps < 0.001), and psychosis and trauma (*p* < 0.05) had significantly longer WASO compared to the reference group. Finally, the estimated sleep efficiency was significantly lower for all diagnostic groups, except for psychosis (see Fig. 1 for details).

## Discussion

The present registry-linked population-based study of older adolescents provides support for sleep problems as a transdiagnostic problem across psychiatric disorders. Sleep disorders that were diagnosed within CAMHS were very rare, with only 0.6% of the adolescents having received a formal sleep disorder diagnosis. However, adolescents with diagnosed psychiatric disorders had significantly higher prevalence of insomnia and DSWPD, and overall most diagnostic groups also had substantially shorter sleep duration, lower sleep efficiency, as well as longer SOL and WASO than the reference group.

Insomnia is highly prevalent both in adolescence [1], and the general adult population [21]. The present study confirms even higher prevalence for adolescents with a psychiatric disorder, with all diagnostic groups presenting with an increased risk. Sleep problems are included as symptoms for a range of psychiatric disorders listed in the DSM-5, and there are also likely common underlying mechanisms, including biological, psychological, and social factors [22]. For instance, for depression, which was the disorder with the highest prevalence of insomnia (48%), these findings correspond well with the established association between depression and sleep problems in adolescence [23].

DSWPD is a much less prevalent condition in the general population, but peaks in late adolescence [24]. The current study provides new evidence of even higher prevalence of DSWPD among adolescents with psychiatric disorders. While the prevalence of DSWPD was low in the reference group at 2.9%, the prevalence among those diagnosed with a psychiatric disorder was higher, with a disorder-specific pattern. Specifically, the highest prevalences were observed among adolescents with conduct disorders at 20%, while there were no identified cases among adolescents with eating disorders. The strong association between DSWPD and conduct disorder and mood disorders is also in line with the pattern of observed oversleeping on weekends among adolescents with these disorders in a US population-based study [4]. Due to the low prevalence, and the related lack of statistical power, we did not perform statistical analysis for the less prevalent psychiatric diagnosis.

Suboptimal sleep patterns and poor sleep quality in general were present across all psychiatric disorders. In particular, short sleep duration caused by long SOL and WASO, was evident. These findings are consistent with the observed short sleep duration during weekdays across diagnostic categories in the above-mentioned US population-based study by Zhang et al. [4]. The clinical significance of the short sleep duration was underscored by the low sleep efficiencies across most diagnostic groups, well below the established clinical cut-off of 85%.

It is noteworthy that the high prevalence of sleep problems as indicated by questionnaires in the epidemiological study was not mirrored by registered diagnosis of sleep disorders in the official registries. The low prevalence of diagnosed sleep problems within CAMHS may not be that surprising, given a similar low prevalence of diagnosed sleep disorders among adults receiving mental health services in Norway [25]. The low prevalence of diagnosed disorders might reflect that sleep assessment and treatment is not integrated into clinical practice, or that while acknowledged, sleep problems are not formalized in patient journals, nor are they seen as severe enough to warrant a diagnosis.

The high prevalence of sleep problems across diagnostic categories supports the central role of sleep problems in psychiatric disorders, which is also central from a transdiagnostic view [16]. Although the present study precludes investigating possible mechanisms involved in these associations, previous studies and models indicate that they share common underlying processes, for instance common genetic influences, biological processes as well as psychological processes [16, 22, 26]. Future studies are needed to understand the mechanism, for instance the strong co-occurrence of DSWPD among adolescents with conduct disorder should be investigated to further elucidate the etiology and pathophysiology.

Some methodological considerations should be noted. The strength of the study is the heterogeneous patient group and clinically diagnosed psychiatric disorders. However, this is was also a weakness of the study, since the clinical assessment may vary across clinicians and is not standardized for the present study. Further, the reference group may include people with mental illness who have not been connected with treatment services. Some of the diagnostic groups were small, such as psychotic disorders, which limits statistical power and the conclusions that can be drawn. The overlap between insomnia and DSWPD was not specifically addressed in the current study, although the overlap is often high [24] and this has important clinical implications. Further, only problems initiating and maintaining sleep were included, and early morning awakenings were not specifically addressed, which is a limitation of the study. Another limitation of the insomnia operalisation is that functional impact was addressed by sleepiness and tiredness, and the perceived impact on other domains was not included. We cannot comment on temporality between the sleep and the psychiatric disorders. However, previous studies have suggested that the link between sleep and specific psychiatric disorders is likely to be bidirectional, with sleep both predicting the development of mental health problems, and impacting treatment [27]. Also, one cannot disregard the possibility that there may be diagnosis-specific directionality, where some diagnoses are bidirectionally associated with sleep, while others have a unidirectional association [7].

## Conclusion

These results confirm the transdiagnostic role of sleep problems in mental disorders in adolescence, emphasizing the importance of thorough sleep assessments which should encompass general sleep patterns and disorders like insomnia and DSWPD. These findings also point to the importance of preventive interventions that could improve sleep among adolescents and might even be one pathway to prevent the development of mental health problems. Finally, interventions for sleep and circadian functioning in youth must become widely available [28]. Tailored transdiagnostic sleep interventions for adolescents that aim at improving sleep and circadian rhythm are good examples of treatments that might be included in the CAMHS [29]. In addition to the expected improvement in sleep problems, they may also give additional benefit for the symptoms of the psychiatric disorders [30, 31].

## Appendix

See Table 3

**Table 3:** Operationalization of broader diagnostic categories in the linked youth@hordaland-NPR sample

Diagnostic category	ICD-10 code	ICD-10 diagnosis
Anxiety disorders	F400	Agoraphobia
	F4000	Agoraphobia
	F401	Social phobia
	F402	Specific (isolated) phobias
	F408	Other phobic anxiety disorders
	F409	Phobic anxiety disorder, unspecified
	F410	Panic disorder [episodic paroxysmal anxiety]
	F411	Generalized anxiety disorder
	F412	Mixed anxiety and depressive disorder
	F413	Other mixed anxiety disorders
	F418	Other specified anxiety disorders
	F419	Anxiety disorder, unspecified
	F420	Predominantly obsessional thoughts or ruminations
	F421	Predominantly compulsive acts [obsessional rituals]
	F422	Mixed obsessional thoughts and acts
	F429	Obsessive–compulsive disorder, unspecified
	F452	Hypochondriacal disorders
	F930	Separation anxiety disorder of childhood
	F931	Phobic anxiety disorder of childhood
	F932	Social anxiety disorder of childhood
	F940	Elective mutism
Mood disorders	F310	Bipolar affective disorder, current episode hypomanic
	F311	Bipolar affective disorder, current episode manic without psychotic symptoms
	F312	Bipolar disorder, current episode manic severe with psychotic features
	F313	Bipolar affective disorder, current episode mild or moderate depression
	F314	Bipolar disorder, current episode depressed, severe, without psychotic features
	F316	Bipolar affective disorder, current episode mixed
	F317	Bipolar affective disorder, currently in remission
	F318	Other bipolar disorders
	F319	Bipolar affective disorder, unspecified
	F320	Mild depressive episode
	F3200	Mild depressive episode without somatic syndrome
	F3201	Mild depressive episode with somatic syndrome
	F321	Moderate depressive episode
	F322	Severe depressive episode without psychotic symptoms
	F323	Major depressive disorder, single episode, severe with psychotic features
	F328	Other depressive episodes
	F329	Depressive episode, unspecified
	F330	Major depressive disorder, recurrent, mild
	F331	Recurrent depressive disorder, current episode moderate
	F3310	Recurrent depressive disorder, current episode moderate, without somatic syndrome
	F332	Recurrent depressive disorder, current episode severe without psychotic symptoms
	F333	Recurrent depressive disorder, current episode severe with psychotic symptoms
	F338	Other recurrent depressive disorders
	F339	Major depressive disorder, recurrent, unspecified
	F341	Dysthymic disorder
	F348	Other persistent mood [affective] disorders
	F349	Persistent mood [affective] disorder, unspecified
	F381	Other recurrent mood [affective] disorders
	F412	Mixed anxiety and depressive disorder
Trauma-related disorders	F430	Acute stress reaction
	F431	Post-traumatic stress disorder
	F4320	Adjustment disorder, unspecified
	F4321	Adjustment disorder with depressed mood
	F4322	Adjustment disorder with anxiety
	F4323	Adjustment disorder with mixed anxiety and depressed mood
	F4324	Adjustment disorder with disturbance of conduct
	F4325	Adjustment disorder with mixed disturbance of emotions and conduct
	F438	Other reactions to severe stress
	F439	Reaction to severe stress, unspecified
Eating disorders	F500	Anorexia nervosa
	F501	Atypical anorexia nervosa
	F503	Atypical bulimia nervosa
	F504	Overeating associated with other psychological disturbances
	F505	Vomiting associated with other psychological disturbances
	F509	Eating disorder, unspecified
Autism-spectrum disorders (ASD)	F840	Childhood autism
	F841	Atypical autism
	F845	Asperger syndrome
	F848	Other pervasive developmental disorders
	F849	Pervasive developmental disorder, unspecified
Conduct disorders	F910	Conduct disorder confined to family context
	F911	Unsocialized conduct disorder
	F912	Conduct disorder, adolescent-onset type
	F913	Oppositional defiant disorder
	F918	Other conduct disorders
	F919	Conduct disorder, unspecified
	F920	Depressive conduct disorder
	F928	Other mixed disorders of conduct and emotions
	F929	Mixed disorder of conduct and emotions, unspecified
Attention-deficit/hyperactivity disorder (ADHD)	F900	Hyperkinetic disorders
	F901	Disturbance of activity and attention
	F908	Other hyperkinetic disorders
	F909	Attention-deficit hyperactivity disorder, unspecified type
Psychotic disorders	F203	Undifferentiated schizophrenia
	F208	Other schizophrenia
	F209	Schizophrenia, unspecified
	F2090	Schizophrenia, unspecified
	F21	Schizotypal disorder
	F231	Acute polymorphic psychotic disorder with symptoms of schizophrenia
	F233	Acute paranoid psychosis without acute stressor
	F239	Acute and transient psychotic disorder, unspecified
	F2390	Acute and transient psychotic disorder, unspecified
	F28	Other psychotic disorder not due to a substance or known physiological condition
	F29	Unspecified nonorganic psychosis
	F312	Bipolar disorder, current episode manic severe with psychotic features
	F323	Major depressive disorder, single episode, severe with psychotic features
	F333	Recurrent depressive disorder, current episode severe with psychotic symptoms
Other axis 1 psychiatric diagnoses	F448	Other dissociative and conversion disorders
	F449	Dissociative [conversion] disorder, unspecified
	F454	Persistent somatoform pain disorder
	F458	Other somatoform disorders
	F480	Neurasthenia
	F489	Neurotic disorder, unspecified
	F510	Nonorganic insomnia
	F512	Nonorganic disorder of the sleep–wake schedule
	F54	Psychological and behavioral factors associated with disorders or diseases classified elsewhere
	F601	Schizoid personality disorder
	F633	Trichotillomania
	F640	Transsexualism
	F659	Disorder of sexual preference, unspecified
	F933	Sibling rivalry disorder
	F938	Other childhood emotional disorders
	F939	Childhood emotional disorder, unspecified
	F941	Reactive attachment disorder of childhood
	F942	Disinhibited attachment disorder of childhood
	F948	Other childhood disorders of social functioning
	F950	Transient tic disorder
	F951	Chronic motor or vocal tic disorder
	F952	Combined vocal and multiple motor tic disorder [de la Tourette]
	F980	Nonorganic enuresis
	F981	Nonorganic encopresis
	F988	Other specified behavioral and emotional disorders with onset usually occurring in childhood and adolescence
	F989	Unspecified behavioral and emotional disorders with onset usually occurring in childhood and adolescence
	F99	Mental disorder, not otherwise specified
	R418	Other symptoms and signs involving cognitive functions and awareness
	R440	Auditory hallucinations
	R441	Visual hallucinations
	R452	Unhappiness
	R454	Irritability and anger
	R455	Hostility
	R457	State of emotional shock and stress, unspecified
	R458	Other symptoms and signs involving emotional state
	R466	Undue concern and preoccupation with stressful events

.
